# Effect of early hospital readmission and comorbid conditions on subsequent long‐term mortality after transient ischemic attack

**DOI:** 10.1002/brb3.865

**Published:** 2017-11-22

**Authors:** Mohammed Yousufuddin, Nathan Young, Lawrence Keenan, Tammy Olson, Jessica Shultz, Taylor Doyle, Eimad Ahmmad, Kogulavadanan Arumaithurai, Paul Takahashi, Mohammad Hassan Murad

**Affiliations:** ^1^ Division of Hospital Medicine Mayo Clinic Health System Austin MN USA; ^2^ Division of Neurology Mayo Clinic Rochester MN USA; ^3^ Division of Cardiology Mayo Clinic Health System Austin MN USA; ^4^ Division of Neurology Mayo Clinic Health System Austin MN USA; ^5^ Division of Primary Care Internal Medicine Mayo Clinic Rochester MN USA; ^6^ Center for the Science of Healthcare Delivery Mayo Clinic Rochester MN USA; ^7^ Division of Preventive Medicine Mayo Clinic Rochester MN USA

**Keywords:** chronic conditions, mortality, Readmission, transient ischemic attack

## Abstract

**Background:**

The implications of early readmission on long‐term mortality after transient ischemic attack (TIA) are not known. We aimed at examining the effect of 180‐day readmission on subsequent long‐term mortality after index hospitalization for TIA.

**Methods:**

A retrospective study of patients hospitalized for first‐ever TIA at Mayo Clinic from 2000 through 2017. Patients readmitted within 180 days postdischarge were compared with those not readmitted in long‐term risk of death.

**Results:**

Of 251 TIA patients aged 73 ± 15 years with 1509 person‐years of follow‐up, 65 (26%) were readmitted within 180 days of discharge and 125 died during a median follow‐up of 5.7 years. The mortality was 10 vs. 7 deaths per 100 person‐years in patients who were readmitted compared to those who were not readmitted with hazard ratio (HR) 1.73 (95% confidence interval [CI] 1.13–2.66). Other competing predictors of mortality were age ≥65 years (HR 5.70, 95% CI 2.72–11.96), cancer (HR 1.65, 95% CI 1.03–3.38), chronic obstructive pulmonary disease (HR 1.90, 95% CI 1.07–3.38), heart failure (HR 3.03, 95% CI 1.82–5.06), dementia (HR 5.87, 95% CI 3.27–10.52), creatinine ≥1.4 mg/dl (HR 1.89, 95% CI 1.17–3.06), and hemoglobin level <10 g/dl (HR 2.80, 95% CI 1.20–6.66).

**Conclusions:**

Hospital readmission within 180 days of discharge from index TIA was associated with increased risk of death several years after initial readmission. Older age and several comorbidities identified during index hospitalization also confer increased risk for long‐term mortality.

## 1 INTRODUCTION

There are an estimated 5 million adults with transient ischemic attack (TIA) in the United States (Benjamin et al., 2017). These patients are at increased of recurrent TIAs, stroke, and death (Clark, Murphy, & Rothwell, 2003; Giles & Rothwell, 2007; Johnston, Gress, Browner, & Sidney, 2000; Wu et al., 2007). Estimates suggest that 15% to 23% of strokes are preceded by a TIA (Johnston et al., 2003; Rothwell & Warlow, 2005). Population‐based studies suggest that TIA patients are especially predisposed to incident stroke and other major cardiovascular events during the immediate postdischarge period (Pendlebury & Rothwell, 2009). The initial period of vulnerability may vary up to 90 days to one year after the initial TIA, during which patients are potentially at heightened risk for major adverse events often necessitating hospitalization (Giles & Rothwell, 2007; Kleindorfer et al., 2005; Wu et al., 2007). Prior studies have largely focused on incident stroke and cardiovascular events and have not examined all‐cause events following index TIA. No studies have examined the relationship between early adverse events following an initial TIA and subsequent long‐term mortality. For instance, in heart failure and chronic obstructive pulmonary disease (COPD), early postdischarge hospital readmission is associated with increased risk of subsequent mortality (Guerrero et al., 2016; Solomon et al., 2007) with no such data after a TIA. Early estimates suggested that the risk of composite of stroke and cardiovascular disease (CVD) events was 12% to 20% within 90 days after index TIA (Giles & Rothwell, 2007; Johnston et al., 2000; Lovett et al., 2003; Pendlebury & Rothwell, 2009). However, a recent report based on a large TIA registry (Amarenco et al., 2016) indicated a decline in the risk of CVD events early after a TIA. Increased prevalence of noncardiovascular comorbidity (Yousufuddin et al., 2017) potentially confers increased risk of subsequent non‐CVD events‐related hospitalization. The implications of comorbid CCs on long‐term prognosis have not been investigated in patients with TIAs.

To address these knowledge gaps, we undertook a retrospective cohort study, primarily, to assess the relationship between 180‐day postdischarge nonfatal hospital readmission from any cause and subsequent long‐term mortality after hospitalization for first‐ever TIA. Our secondary objective was to examine the influence of comorbid CCs, identified at the time of diagnosis, on the long‐term mortality after an initial TIA.

## 2 METHODS

### 2.1 Study design and population

This is a retrospective study of patients aged ≥18 years, hospitalized for first‐ever TIA at Mayo Clinic, followed up until death or censored on May 30, 2017. The look back period extended to 5 years to ensure accuracy of a first‐ever event. The initial cohort of hospitalized patients with TIA was identified using *International Classification of Diseases, Ninth Revision, Clinical Modification* (ICD‐9‐CM) codes. Previous studies have demonstrated a high positive predictive value of ICD‐9‐CM codes for the discharge diagnoses of stroke and TIA (Kokotailo & Hill, 2005; Tirschwell & Longstreth, 2002). Subsequently, three study investigators reviewed electronic health records of individual study patients to collect data related to demographics, clinical features, vital signs, primary and secondary diagnoses, investigations, procedures, repeat hospitalizations, and death. To ensure completeness of readmissions following discharge from index hospitalization, only patients from Olmsted County were enrolled in the study and those from other counties were excluded.

### 2.2 Definition of transient ischemic attack

We incorporated both time‐ and tissue‐based definitions for the purpose of enrollment of patient in this study. Accordingly, TIA was defined as a brief episode of neurological dysfunction caused by focal brain or retinal ischemia with clinical symptoms lasting <24 hr and without radiological evidence of acute infarction (Easton et al., 2009). Patients who had residual or persistent deficit beyond 24 hr or who demonstrated radiological evidence of acute infarction were excluded from the study. Patients with a previous history of stroke or TIA documented in medical records, hypertensive encephalopathy, reversible cerebral vasoconstriction syndrome, diagnostic uncertainty, on dialysis, on a transplant list, organ transplant recipients, or recipients of left ventricular assist device were all excluded as their mortality was less likely to be influenced by index TIA event. We used Trial of Org 10172 in Acute Stroke Treatment (TOAST) classification system to estimate the presumed mechanism of TIA. According to this scheme the five main mutually exclusive categories were as follows: 1) large artery atherosclerosis, 2) cardio‐embolism, 3) small vessel occlusion, 4) other determined cause, and 5) undetermined cause (Adams et al., 1993). Probable TIA territory was classified either anterior or posterior circulations based on predominant symptoms on admission and as reported by previous studies (Dornak et al., 2015; Nouh, Remke, & Ruland, 2014). Electronic medical record of each patient was critically reviewed to ensure appropriate assignment.

### 2.3 Measures of chronic conditions

Comorbid CCs were identified at the time of index hospitalization. A chronic condition is defined as long‐term conditions requiring medical attention (Goodman, Posner, Huang, Parekh, & Koh, 2013). Initially we selected 20 CCs specified by the Office of the Assistant Secretary for Health. Since cerebrovascular disease is the focus of this study, stroke was excluded from the list of 20 CCs. The presence of the remaining 19 conditions in our cohort was identified using Clinical Classifications Software (CCS) code that group ICD‐9‐CM or ICD‐10 codes and are part of US Health Care Cost and Utilization project (H‐CUP) tool. CCs with frequency of <5% were excluded from the analysis.

### 2.4 Measures of outcomes

Readmissions were defined as the occurrence of one or more repeat hospitalizations from any cause within 180 days of discharge from index hospitalization for TIA. Readmissions were stratified according to cause as CVD or non‐CVD event. Long‐term mortality was defined as death from any cause among survivors to 180 days of discharge from index hospitalization up until May 30, 2017.

### 2.5 Statistical analysis

Baseline characteristics were reported as mean and standard deviation (SD) for continuous variables and whole numbers and percentages for categorical variables. Study groups were compared for continuous variables using the two‐tailed, unpaired, Student's *t‐*test, and categorical variables using Pearson χ^2^ test. Survival analysis was performed using Kaplan–Meier curves with death as the outcome and groups with and without early readmission were compared using the log‐rank test. The effect of hospital readmission from any cause within 180 days on the risk of death was determined by Cox proportional hazard regression analyses adjusted to multiple variables. The covariates for multivariable adjustment were prespecified and all included in model development. The Cox proportional hazard regression model was performed through stepwise selection with inclusion set at *p *= .1 and exclusion set at *p* > .05. The candidate predictors incorporated were as follows: age <65 years (reference) and ≥65 year, BMI <30 (reference) and ≥30 kg/m^2^; tobacco use: current smoker, ex‐smoker, and never smoker (reference), SBP <140 mm Hg (reference) and ≥140 mmHg, hemoglobin <10 g/dl and ≥10 g/dl (reference), creatinine <1.4 mg/dl (reference group) and ≥1.4 m/dl, HDL cholesterol ≥50 mg/dl (reference) and <50 mg/dl, LDL cholesterol <100 mg/dl (reference) and ≥100 mg /dl, drugs on dismissal: antiplatelet (reference) vs. no antiplatelet, antihypertensive vs. no antihypertensive (reference), statin vs. no statin (reference), presumed mechanism of TIA: large artery atherosclerosis, cardio‐embolism, small vessel occlusion, other determined cause, and undermined cause (reference), anterior circulation TIA (reference) vs. posterior circulation TIA. We performed multiple sensitivity analyses to investigate the effect of: 1) CVD event‐related hospital readmission as compared to non‐CVD event‐related readmission and 2) disease‐specific (stroke and TIA) incident readmission. Patients were censored until the time of death or study closure date of May 30, 2017, whichever occurred first. The statistical analyses were performed using JMP pro 10 version and Statistical Analysis System (SAS Institute) version 10. For all analyses, a two‐side *p *<* *.05 was considered statistically significant.

## 3 RESULTS

### 3.1 Patient characteristics

**Figure 1:**
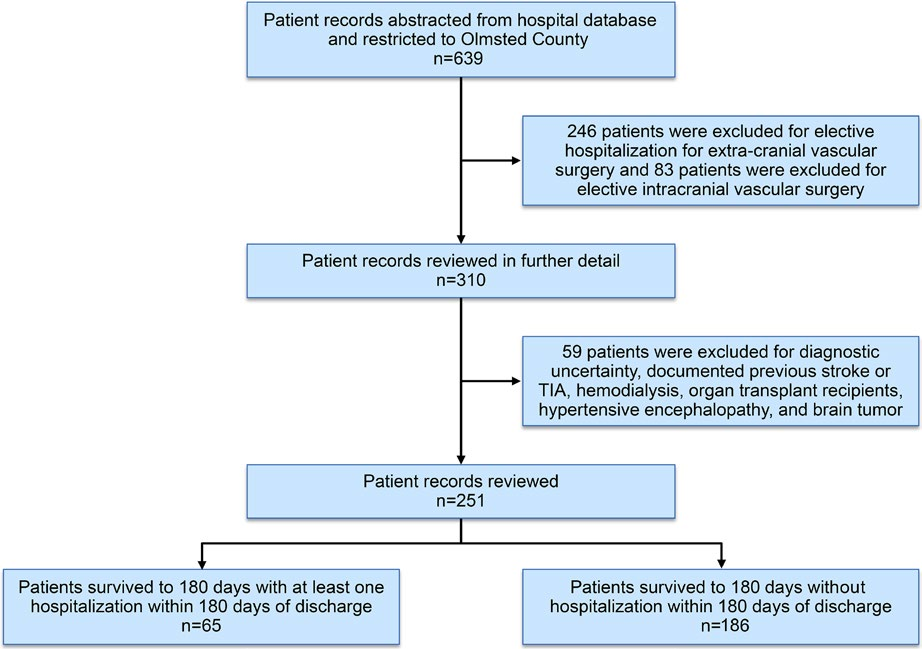
Study flow diagram: selection of study cohort

Figure 1 describes the patient selection and enrollment process from data extraction to identification of study cohort and stratification of study groups according to readmission within 180 days of discharge from index TIA. The final TIA cohort comprised of 251 adults, all from Olmsted County, Minnesota, USA. Of these, 182 (72%) were admitted to a specialist stroke service. The remaining 69 (28%) patients who were admitted to other services were managed in consultation with a neurologist throughout the course of their hospitalization. The baseline characteristics of the study population, stratified by hospital readmission and no readmission within 180 days are summarized in Table 1. Patients who had readmission and those did not have readmission within 180 days were comparable in all baseline characteristics except for greater frequency of anemia and more often performance of transthoracic echocardiogram/transesophageal echocardiogram in the former than the latter group. For the entire cohort, the mean age was 73.1 ± 14.8 (standard deviation SD) years, 51% were men, and 95% were non‐Hispanic whites.

**Table 1:** Baseline characteristics stratified by the presence or absence of hospital readmission within 180 days of index hospitalization for transient ischemic attack

	Hospital readmission within 180 days (*n *= 65) (%)	No hospital readmission within 180 days (*n *= 186) (%)	*p* value
Demographics			
Age	73.8 ± 16.0	72.8 ± 14.5	.64
Male	36 (55)	99 (53)	.23
Whites	60 (92)	177 (95)	.38
Admission type			
Stroke Service	49 (75)	133 (71)	.55
LOS (days)	2.4 ± 2.5	2.2 ± 1.6	.27
Tobacco smoking			
Current	9 (14)	24 (13)	.84
Past	21 (32)	67 (36)	.59
Never	33 (51)	90 (48)	.74
Blood pressure			
SBP (mmHg)	145 ± 24	151 ± 30	.29
DBP (mmHg)	76 ± 15	78 ± 20	.38
PP (mmHg)	70 ± 20	72 ± 25	.51
Anthropometry			
BMI (Kg/m^2^)	26.8 ± 5.3	28.2 ± 5.9	.11
Key laboratory findings			
Hb (g/dl)	12.6 ± 1.6	13.9 ± 9.0	.24
BUN (mg/dl)	20.3 ± 9.6	21.3 ± 14.0	.58
Creatinine (mg/dl)	1.1 ± 0.4	1.1 ± 0.3	.81
HDL‐C (mg/dl)	49.8 ± 15.4	49.7 ± 14.5	.98
LDL‐C (mg/dl)	94.5 ± 34.4	100.4 ± 35.9	.28
Drugs			
Antiplatelets	57 (90)	165 (90)	.89
Warfarin	14 (21)	34 (18)	.55
Statin	30 (47)	102 (55)	.24
Beta‐blocker	28 (44)	84 (45)	.79
ACEI/ARBS	28 (44)	67 (30)	.29
Prevalent comorbid chronic conditions			
Dyslipidemia	33 (50)	91 (49)	.79
Hypertension	47 (72)	143 (77)	.46
Depression	16 (25)	32 (17)	.19
Diabetes	11 (17)	38 (20)	.53
Arthritis	19 (29)	59 (31)	.70
Cancer	11 (17)	28 (15)	.72
Atrial fibrillation	13 (20)	42 (23)	.66
CAD	24 (36)	56 (30)	.31
COPD	5 (7)	18 (10)	.63
Osteoporosis	8 (12)	20 (11)	.73
CKD	7 (11)	24 (13)	.65
Heart Failure	10 (15)	19 (10)	.26
Dementia	4 (6)	18 (10)	.39
Anemia	25 (38)	37 (20)	.003
Imaging studies			
CT‐head	62 (95)	177 (95)	.94
CTA‐head & neck	8 (12)	11 (5)	.09
MRI‐brain	43 (66)	120 (64)	.81
MRA‐head and neck	35 (54)	86 (47)	.29
Carotid Duplex US	46 (71)	131 (70)	.95
TTE/TEE	51 (80)	112 (64)	.01
Probable mechanism of TIA			
Large artery atherosclerosis	11 (17)	29 (15)	.80
Cardio‐embolism	17 (26)	35 (19)	.21
Small vessel occlusion	13 (20)	49 (26)	.31
Other determined cause	2 (3)	6 (3)	.95
Undermined cause	22 (34)	67 (36)	.75
Probable localization of arterial territory			
Anterior circulation	52 (80)	146 (78)	.79
Posterior circulation	14 (20)	43 (22)	.79

ACEI/ARBS, angiotensin‐converting enzyme inhibitor/ angiotensin II receptor blocker; BMI, body mass index; BUN, blood urea nitrogen; CAD, coronary artery disease; CKD, chronic kidney disease; CT, computed tomography; CTA, computed tomographic angiogram; COPD, chronic obstructive pulmonary disease; DBP, diastolic blood pressure; Hb, hemoglobin; HDL‐C, high‐density lipoprotein cholesterol; LDL‐C, low‐density lipoprotein cholesterol; LOS, length of stay; MRI, magnetic resonance imaging; MRA, magnetic resonance angiography; PP, pulse pressure; TIA, transient ischemic attack; SBP, systolic blood pressure; TTE/TEE, transthoracic echo/transesophageal echo.

### 3.2 Hospital readmission

All‐cause nonfatal repeat hospitalizations within 180 days of discharge occurred in 65 (26%) patients including 37 (57%) from non‐CVD conditions and 28 (43%) from CVD conditions (recurrent TIAs 7 [11%], incident strokes 8 [12%], other cardiovascular conditions including planned readmissions 13 [20%]).

### 3.3 Mortality

During 1509 person‐years of follow‐up (median 5.74 years, range 0.34–16.17 years) a total of 125 patients (48%) died. Of 125 deaths, 5(2%) occurred within first 180 days of discharge including 2 (3%) deaths among patients readmitted within 180 days and 3 (1.5%) deaths among those not readmitted within 180 days. The post 180‐day mortality rate was 10 deaths per 100 person‐years in readmission group (cumulative mortality: 36 patients, 55%) in readmission cohort and 7 deaths per 100 person‐years (cumulative mortality: 84 patients, 45%) in no readmission cohort.

### 3.4 Effect of hospital readmission and other factors on mortality

**Figure 2:**
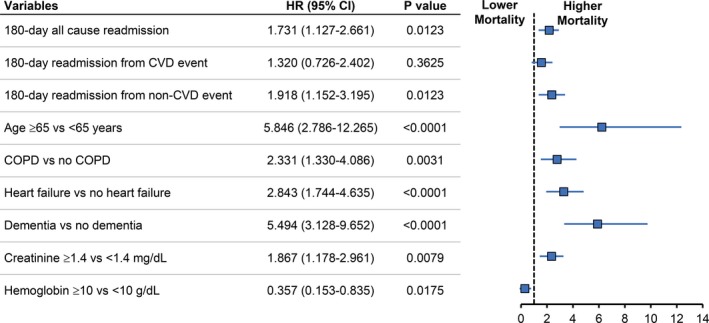
Multivariate Cox proportional hazard regression analysis and forest plot. CI, confidence interval; COPD, chronic obstructive pulmonary disease; CVD, cardiovascular disease; HR, hazard ratio

The unadjusted Kaplan–Meier estimate of long‐term mortality demonstrated a trend toward increased event rate among TIA patients who were readmitted within 180 days of discharge compared with the other group (*p* = .11 log‐rank test, 0.09 Wilcoxon). The results were not affected when readmissions were stratified according to cause as CVD and non‐CVD events. In the Cox proportional hazard model with multivariable adjustments, the mortality was significantly increased with the adjusted hazard ratio (HR) of 1.73 (95% confidence interval [CI] 1.13–2.66) in the cohort with 180‐day readmission compared with the cohort with no readmission. Additionally, age ≥65 years (HR 5.70, 95% CI 2.72–11.96) and comorbid cancer (HR 1.65, 95% CI 1.03–3.38), COPD (HR 1.90, 95% CI 1.07–3.38), heart failure (HR 3.03, 95% CI 1.82–5.06), dementia (HR 5.87, 95% CI 3.27–10.52), creatinine ≥ 1.4 mg/dl (HR 1.89, 95% CI 1.17–3.06), and hemoglobin level <10 g/dl (HR 1.60, 95% CI 0.06–0.83) have emerged as independent predictors of increased risk of death in patients with TIA. The Results were depicted in Figure 2.

### 3.5 Sensitivity analysis

Because the majority of hospitalizations within 180 days of discharge were from non‐CVD conditions (57%), we performed sensitivity analysis for mortality after excluding patients who were hospitalized for cardiovascular conditions (43%). The overall, cumulative risk of repeat hospitalization for incident stroke was 11% (*n *= 27) and incident recurrent TIA was 3% (8%) until end of follow‐up. These analyses did not alter the results substantially.

## 4 DISCUSSION

To the best of our knowledge, this is the first study to examine the relationship between early nonfatal hospital readmission after index hospitalization with first‐ever TIA and subsequent long‐term death. Previously published mortality and incident stroke estimates following TIA were largely centered on ≤90‐day follow up time point (Giles & Rothwell, 2007; Wu et al., 2007). Because of anticipated low event rate in our study, we chose 180 days, which still represent an early risk given the natural history of TIAs, as the follow‐up time point to estimate early hospital readmissions. We found that approximately one in four patients with an initial TIA were rehospitalized within 180 days of discharge, predominantly from non‐CVD events, a finding consistent with published studies in heart failure (Krumholz et al., 1997) and other non‐TIA conditions (Dharmarajan et al., 2013; Jencks, Williams, & Coleman, 2009).

The main finding that emerged from this study was that hospital readmission during the first 180 days of a TIA was associated with a 1.7 fold increase in the risk of subsequent all‐cause death, compared with those with no 180‐day hospital readmission. The clinical impact of 180‐day hospital readmission on long‐term mortality was independent of etiology and numerous other candidate predictors. There are no comparable clinical trials, specifically, in TIA, but the data from previous studies in stroke (Bravata, Ho, Meehan, Brass, & Concato, 2007) and several other nonstroke and non‐TIA conditions such as heart failure (Solomon et al., 2007), acute myocardial infarction (Abrahamsson et al., 2009), and COPD (Guerrero et al., 2016; Soler‐Cataluna et al., 2005) are broadly in concordance with our findings. Hospital readmission in the course of heart failure, acute myocardial infarction, and COPD potentially represents a sentinel event and confer a high risk for subsequent mortality (Genao et al., 2015; Giamouzis et al., 2011; Kociol et al., 2010). On the other hand a TIA is generally considered to be a reversible condition with variable, but, relatively low excess mortality (Clark et al., 2003; Grass, 1988; van Wijk et al., 2005). Nevertheless, TIAs with or without concomitant chronic condition(s), may have the potential to trigger a similar level of acute neuro‐hormonal and autonomic stress that potentially results in hospital readmission from any cause and consequent increased mortality. The patient population in our study were those with a first‐ever TIA. On the contrary, studies examining the impact of hospital readmission on subsequent mortality in patients with heart failure and COPD variably incorporated patients with prior hospitalizations for acute decompensation, thereby confounding the inferential analyses (Guerrero et al., 2016; Solomon et al., 2007). Contradicting our finding in TIAs, a recent report by Krumholz et al., in patients with acute myocardial infarction and pneumonia, suggested that hospital readmission and mortality represent two distinct measures with differential information and showed no association between hospital readmission and mortality (Krumholz et al., 2013). The differences may be attributable to patient characteristics, study design and length of follow‐up.

It was not surprising that we identified a number of other variables as independent predictors of long‐term mortality across a broad spectrum of TIA patients regardless of presumed mechanisms of TIA, vascular territory affected, length of hospital stay, or therapeutic strategies on dismissal. These findings were valid even accounting for sex, smoking status, body mass index, systolic blood pressure, diastolic blood pressure, pulse pressure, HDL cholesterol, and LDL cholesterol. We found that the age, as anticipated, was a strong predictor of mortality in patients aged ≥65 years and were at a 5.9‐fold greater risk of death than those aged <65 years after a first‐ever TIA, in concordance with a previously published study (van Wijk et al., 2005). The prevalence of heart failure, dementia, stroke, chronic kidney disease, and anemia are common among patients with stroke and confer increased mortality (Barba et al., 2002; Desmond, Moroney, Sano, & Stern, 2002; Divani, Vazquez, Asadollahi, Qureshi, & Pullicino, 2009; Lee et al., 2017; Sharma, Fletcher, Vassallo, & Ross, 2000; Strachan, 1991; Wannamethee, Shaper, & Ebrahim, 1995). We recently reported that a number of comorbid CCs were associated with 30‐day postdischarge mortality in patients with initial stroke and TIA (Yousufuddin et al., 2017). In this study, we extended these prior findings to predict the long‐term mortality in patients with a first‐ever TIA and demonstrated that comorbid heart failure (2.8‐fold), dementia (5.5‐fold), COPD (2.3‐fold), anemia with hemoglobin level <10 g/dl (1.6‐fold), CKD with creatinine ≥1.4 mg/dl (1.9) were all independent predictors of increased risk of long‐term death after index hospitalization for a TIA. These finding have important clinical implication for targeting long‐term mortality after an initial TIA. We failed to find a discernible relationship between mortality after an initial TIA and other select characteristics including vascular territory of the TIA, etiological subtype, current tobacco smoking, systolic blood pressure, levels of HDL‐ and LDL‐cholesterol on admission, inconsistent with previous data linking such relationship (Amarenco et al., 2016; Flossmann & Rothwell, 2003; Heart Protection Study Collaborative G, 2002; Howard, Toole, Frye‐Pierson, & Hinshelwood, 1987; Johnston et al., 2007; Lovett, Coull, & Rothwell, 2004), potentially explained by differences in patient characteristics and study design.

We acknowledge several limitations and strengths in this study. The study population is biased toward specialized stroke services and may not represent TIA patients in the community presenting to general medical clinics and emergency departments. Although, sample size is relatively small, follow‐up is sufficiently long and the number of events provided adequate precision allowing us to derive reasonable conclusions. The strengths of this study include well characterized patient population with regards to primary and secondary diagnoses, relatively complete follow‐up evidenced by review of data from electronic medical records on or shortly before censor May 30, 2017. Furthermore, patients were reliably classified into etiological subgroups based on imaging studies and medical records documentation. The electronic medical record system is one of the oldest in the country and with documented efficiency and a high level of case ascertainment for incident TIA and prompt mortality updates (Whisnant et al., 1990).

In summary, we found that early hospitalizations following initial TIA were common and were associated with increased risk of subsequent longer‐term mortality. Additionally, age and selected comorbid chronic conditions including heart failure, COPD, dementia, anemia, and CKD were all independent predictors of increased risk of subsequent death after initial TIA. These findings underscore the importance of all‐cause early postdischarge readmissions and suggest the need for developing therapeutic strategies beyond preventing stroke and other CVD events to improve overall long‐term mortality after initial TIA. Knowledge of these prognostic variables can help healthcare providers tailor post‐discharge care and identify comorbidities to be addressed in the outpatient setting. It can also empower patients with appropriate prognostic information for healthcare needs.

## CONFLICTS OF INTEREST

The authors whose names are listed immediately below certify that they have NO affiliations with or involvement in any organization or entity with any financial interest (such as honoraria; educational grants; participation in speakers’ bureaus; membership, employment, consultancies, stock ownership, or other equity interest; and expert testimony or patent‐licensing arrangements), or nonfinancial interest (such as personal or professional relationships, affiliations, knowledge or beliefs) in the subject matter or materials discussed in this manuscript.

## References

[brb3865-bib-0001] Abrahamsson, P. , Dobson, J. , Granger, C. B. , McMurray, J. J. , Michelson, E. L. , Pfeffer, M. , … Investigators, C. (2009). Impact of hospitalization for acute coronary events on subsequent mortality in patients with chronic heart failure. European Heart Journal, 30, 338–345. 19001475 10.1093/eurheartj/ehn503

[brb3865-bib-0002] Adams, H. P. Jr , Bendixen, B. H. , Kappelle, L. J. , Biller, J. , Love, B. B. , Gordon, D. L. , & Marsh, E. E. 3rd (1993). Classification of subtype of acute ischemic stroke. Definitions for use in a multicenter clinical trial. TOAST. Trial of Org 10172 in Acute Stroke Treatment. Stroke, 24, 35–41. 7678184 10.1161/01.str.24.1.35

[brb3865-bib-0003] Amarenco, P. , Lavallee, P. C. , Labreuche, J. , Albers, G. W. , Bornstein, N. M. , Canhao, P. , … Wong LK and Investigators TIo (2016). One‐year risk of stroke after transient ischemic attack or minor stroke. New England Journal of Medicine, 374, 1533–1542. 27096581 10.1056/NEJMoa1412981

[brb3865-bib-0004] Barba, R. , Morin, M. D. , Cemillan, C. , Delgado, C. , Domingo, J. , & Del Ser, T. (2002). Previous and incident dementia as risk factors for mortality in stroke patients. Stroke, 33, 1993–1998. 12154251 10.1161/01.str.0000017285.73172.91

[brb3865-bib-0005] Benjamin, E. J. , Blaha, M. J. , Chiuve, S. E. , Cushman, M. , Das, S. R. , Deo, R. , … American Heart Association Statistics C and Stroke Statistics S (2017). Heart disease and stroke statistics‐2017 update: A report from the American Heart Association. Circulation, 135, e146–e603. 28122885 10.1161/CIR.0000000000000485 PMC5408160

[brb3865-bib-0006] Bravata, D. M. , Ho, S. Y. , Meehan, T. P. , Brass, L. M. , & Concato, J. (2007). Readmission and death after hospitalization for acute ischemic stroke: 5‐year follow‐up in the medicare population. Stroke, 38, 1899–1904. 17510453 10.1161/STROKEAHA.106.481465

[brb3865-bib-0007] Clark, T. G. , Murphy, M. F. , & Rothwell, P. M. (2003). Long term risks of stroke, myocardial infarction, and vascular death in “low risk” patients with a non‐recent transient ischaemic attack. Journal of Neurology, Neurosurgery, and Psychiatry., 74, 577–580. 10.1136/jnnp.74.5.577 PMC1738460 12700296

[brb3865-bib-0008] Desmond, D. W. , Moroney, J. T. , Sano, M. , & Stern, Y. (2002). Mortality in patients with dementia after ischemic stroke. Neurology, 59, 537–543. 12196645 10.1212/wnl.59.4.537

[brb3865-bib-0009] Dharmarajan, K. , Hsieh, A. F. , Lin, Z. , Bueno, H. , Ross, J. S. , Horwitz, L. I. , … Krumholz, H. M. (2013). Diagnoses and timing of 30‐day readmissions after hospitalization for heart failure, acute myocardial infarction, or pneumonia. JAMA, 309, 355–363. 23340637 10.1001/jama.2012.216476 PMC3688083

[brb3865-bib-0010] Divani, A. A. , Vazquez, G. , Asadollahi, M. , Qureshi, A. I. , & Pullicino, P. (2009). Nationwide frequency and association of heart failure on stroke outcomes in the United States. J Card Fail., 15, 11–16. 19181288 10.1016/j.cardfail.2008.09.001

[brb3865-bib-0011] Dornak, T. , Kral, M. , Hazlinger, M. , Herzig, R. , Veverka, T. , Burval, S. , … Kanovsky, P. (2015). Posterior vs. anterior circulation infarction: Demography, outcomes, and frequency of hemorrhage after thrombolysis. International Journal of Stroke, 10, 1224–1228. 26310390 10.1111/ijs.12626

[brb3865-bib-0012] Easton, J. D. , Saver, J. L. , Albers, G. W. , Alberts, M. J. , Chaturvedi, S. , Feldmann, E. , … American Heart A, American Stroke Association Stroke C, Council on Cardiovascular S, Anesthesia, Council on Cardiovascular R, Intervention, Council on Cardiovascular N and Interdisciplinary Council on Peripheral Vascular D (2009). Definition and evaluation of transient ischemic attack: A scientific statement for healthcare professionals from the American Heart Association/American Stroke Association Stroke Council; Council on Cardiovascular Surgery and Anesthesia; Council on Cardiovascular Radiology and Intervention; Council on Cardiovascular Nursing; and the Interdisciplinary Council on Peripheral Vascular Disease. The American Academy of Neurology affirms the value of this statement as an educational tool for neurologists. Stroke, 40, 2276–2293. 19423857 10.1161/STROKEAHA.108.192218

[brb3865-bib-0013] Flossmann, E. , & Rothwell, P. M. (2003). Prognosis of vertebrobasilar transient ischaemic attack and minor stroke. Brain, 126, 1940–1954. 12847074 10.1093/brain/awg197

[brb3865-bib-0014] Genao, L. , Durheim, M. T. , Mi, X. , Todd, J. L. , Whitson, H. E. , & Curtis, L. H. (2015). Early and long‐term outcomes of older adults after acute care encounters for chronic obstructive pulmonary disease exacerbation. Annals of the American Thoracic Society, 12, 1805–1812. 26394180 10.1513/AnnalsATS.201504-250OC PMC4722826

[brb3865-bib-0015] Giamouzis, G. , Kalogeropoulos, A. , Georgiopoulou, V. , Laskar, S. , Smith, A. L. , Dunbar, S. , … Butler, J. (2011). Hospitalization epidemic in patients with heart failure: Risk factors, risk prediction, knowledge gaps, and future directions. Journal of Cardiac Failure, 17, 54–75. 21187265 10.1016/j.cardfail.2010.08.010

[brb3865-bib-0016] Giles, M. F. , & Rothwell, P. M. (2007). Risk of stroke early after transient ischaemic attack: A systematic review and meta‐analysis. Lancet Neurology, 6, 1063–1072. 17993293 10.1016/S1474-4422(07)70274-0

[brb3865-bib-0017] Goodman, R. A. , Posner, S. F. , Huang, E. S. , Parekh, A. K. , & Koh, H. K. (2013). Defining and measuring chronic conditions: Imperatives for research, policy, program, and practice. Preventing Chronic Disease, 10, E66. 23618546 10.5888/pcd10.120239 PMC3652713

[brb3865-bib-0018] Grass, JA (1988). Patient‐controlled analgesia. JAMA, 259, 2240. 3352117

[brb3865-bib-0019] Guerrero, M. , Crisafulli, E. , Liapikou, A. , Huerta, A. , Gabarrus, A. , Chetta, A. , … Torres, A. (2016). Readmission for acute exacerbation within 30 days of discharge is associated with a subsequent progressive increase in mortality risk in COPD patients: A long‐term observational study. PLoS ONE, 11, e0150737. 26943928 10.1371/journal.pone.0150737 PMC4778849

[brb3865-bib-0020] Heart Protection Study Collaborative G (2002). MRC/BHF Heart Protection Study of cholesterol lowering with simvastatin in 20,536 high‐risk individuals: A randomised placebo‐controlled trial. Lancet, 360, 7–22. 12114036 10.1016/S0140-6736(02)09327-3

[brb3865-bib-0021] Howard, G. , Toole, J. F. , Frye‐Pierson, J. , & Hinshelwood, L. C. (1987). Factors influencing the survival of 451 transient ischemic attack patients. Stroke, 18, 552–557. 3590245 10.1161/01.str.18.3.552

[brb3865-bib-0022] Jencks, S. F. , Williams, M. V. , & Coleman, E. A. (2009). Rehospitalizations among patients in the Medicare fee‐for‐service program. New England Journal of Medicine, 360, 1418–1428. 19339721 10.1056/NEJMsa0803563

[brb3865-bib-0023] Johnston, S. C. , Fayad, P. B. , Gorelick, P. B. , Hanley, D. F. , Shwayder, P. , van Husen, D. , & Weiskopf, T. (2003). Prevalence and knowledge of transient ischemic attack among US adults. Neurology, 60, 1429–1434. 12743226 10.1212/01.wnl.0000063309.41867.0f

[brb3865-bib-0024] Johnston, S. C. , Gress, D. R. , Browner, W. S. , & Sidney, S. (2000). Short‐term prognosis after emergency department diagnosis of TIA. JAMA, 284, 2901–2906. 11147987 10.1001/jama.284.22.2901

[brb3865-bib-0025] Johnston, S. C. , Rothwell, P. M. , Nguyen‐Huynh, M. N. , Giles, M. F. , Elkins, J. S. , Bernstein, A. L. , & Sidney, S. (2007). Validation and refinement of scores to predict very early stroke risk after transient ischaemic attack. Lancet, 369, 283–292. 17258668 10.1016/S0140-6736(07)60150-0

[brb3865-bib-0026] Kleindorfer, D. , Panagos, P. , Pancioli, A. , Khoury, J. , Kissela, B. , Woo, D. , … Broderick, J. P. (2005). Incidence and short‐term prognosis of transient ischemic attack in a population‐based study. Stroke, 36, 720–723. 15731465 10.1161/01.STR.0000158917.59233.b7

[brb3865-bib-0027] Kociol, R. D. , Hammill, B. G. , Fonarow, G. C. , Klaskala, W. , Mills, R. M. , Hernandez, A. F. , & Curtis, L. H. (2010). Generalizability and longitudinal outcomes of a national heart failure clinical registry: Comparison of Acute Decompensated Heart Failure National Registry (ADHERE) and non‐ADHERE Medicare beneficiaries. American Heart Journal, 160, 885–892. 21095276 10.1016/j.ahj.2010.07.020

[brb3865-bib-0028] Kokotailo, R. A. , & Hill, M. D. (2005). Coding of stroke and stroke risk factors using international classification of diseases, revisions 9 and 10. Stroke, 36, 1776–1781. 16020772 10.1161/01.STR.0000174293.17959.a1

[brb3865-bib-0029] Krumholz, H. M. , Lin, Z. , Keenan, P. S. , Chen, J. , Ross, J. S. , Drye, E. E. , … Normand, S. L. (2013). Relationship between hospital readmission and mortality rates for patients hospitalized with acute myocardial infarction, heart failure, or pneumonia. JAMA, 309, 587–593. 23403683 10.1001/jama.2013.333 PMC3621028

[brb3865-bib-0030] Krumholz, H. M. , Parent, E. M. , Tu, N. , Vaccarino, V. , Wang, Y. , Radford, M. J. , & Hennen, J. (1997). Readmission after hospitalization for congestive heart failure among Medicare beneficiaries. Archives of Internal Medicine, 157, 99–104. 8996046

[brb3865-bib-0031] Lee, M. J. , Chung, J. W. , Ahn, M. J. , Kim, S. , Seok, J. M. , Jang, H. M. , … Bang, O. Y. (2017). Hypercoagulability and mortality of patients with stroke and active cancer: The OASIS‐CANCER Study. J Stroke., 19, 77–87. 28030894 10.5853/jos.2016.00570 PMC5307941

[brb3865-bib-0032] Lovett, J. K. , Coull, A. J. , & Rothwell, P. M. (2004). Early risk of recurrence by subtype of ischemic stroke in population‐based incidence studies. Neurology, 62, 569–573. 14981172 10.1212/01.wnl.0000110311.09970.83

[brb3865-bib-0033] Lovett, J. K. , Dennis, M. S. , Sandercock, P. A. , Bamford, J. , Warlow, C. P. , & Rothwell, P. M. (2003). Very early risk of stroke after a first transient ischemic attack. Stroke, 34, e138–e140. 12855835 10.1161/01.STR.0000080935.01264.91

[brb3865-bib-0034] Nouh, A. , Remke, J. , & Ruland, S. (2014). Ischemic posterior circulation stroke: A review of anatomy, clinical presentations, diagnosis, and current management. Front Neurology, 5, 30. 10.3389/fneur.2014.00030 PMC3985033 24778625

[brb3865-bib-0035] Pendlebury, S. T. , & Rothwell, P. M. (2009). Risk of recurrent stroke, other vascular events and dementia after transient ischaemic attack and stroke. Cerebrovascular Disease, 27(Suppl 3), 1–11. 10.1159/000209260 19439935

[brb3865-bib-0036] Rothwell, P. M. , & Warlow, C. P. (2005). Timing of TIAs preceding stroke: Time window for prevention is very short. Neurology, 64, 817–820. 15753415 10.1212/01.WNL.0000152985.32732.EE

[brb3865-bib-0037] Sharma, J. C. , Fletcher, S. , Vassallo, M. , & Ross, I. (2000). Cardiovascular disease and outcome of acute stroke: Influence of pre‐existing cardiac failure. European Journal of Heart Failure, 2, 145–150. 10856727 10.1016/s1388-9842(00)00067-2

[brb3865-bib-0038] Soler‐Cataluna, J. J. , Martinez‐Garcia, M. A. , Roman Sanchez, P. , Salcedo, E. , Navarro, M. , & Ochando, R. (2005). Severe acute exacerbations and mortality in patients with chronic obstructive pulmonary disease. Thorax, 60, 925–931. 16055622 10.1136/thx.2005.040527 PMC1747235

[brb3865-bib-0039] Solomon, S. D. , Dobson, J. , Pocock, S. , Skali, H. , McMurray, J. J. , Granger, C. B. , … Pfeffer, M. A. (2007). Candesartan in Heart failure: Assessment of Reduction in M and morbidity I. Influence of nonfatal hospitalization for heart failure on subsequent mortality in patients with chronic heart failure. Circulation, 116, 1482–1487. 17724259 10.1161/CIRCULATIONAHA.107.696906

[brb3865-bib-0040] Strachan, D. P. (1991). Ventilatory function as a predictor of fatal stroke. BMJ, 302, 84–87. 1995121 10.1136/bmj.302.6768.84 PMC1668906

[brb3865-bib-0041] Tirschwell, D. L. , & Longstreth, W. T. Jr (2002). Validating administrative data in stroke research. Stroke, 33, 2465–2470. 12364739 10.1161/01.str.0000032240.28636.bd

[brb3865-bib-0042] Wannamethee, S. G. , Shaper, A. G. , & Ebrahim, S. (1995). Respiratory function and risk of stroke. Stroke, 26, 2004–2010. 7482639 10.1161/01.str.26.11.2004

[brb3865-bib-0043] Whisnant, J. P. , Melton, L. J. 3rd , Davis, P. H. , O'Fallon, W. M. , Nishimaru, K. , & Schoenberg, B. S. (1990). Comparison of case ascertainment by medical record linkage and cohort follow‐up to determine incidence rates for transient ischemic attacks and stroke. Journal of Clinical Epidemiology, 43, 791–797. 2384767 10.1016/0895-4356(90)90239-l

[brb3865-bib-0044] van Wijk, I. , Kappelle, L. J. , van Gijn, J. , Koudstaal, P. J. , Franke, C. L. , Vermeulen, M. , … Li LACsg (2005). Long‐term survival and vascular event risk after transient ischaemic attack or minor ischaemic stroke: A cohort study. Lancet, 365, 2098–2104. 15964446 10.1016/S0140-6736(05)66734-7

[brb3865-bib-0045] Wu, C. M. , McLaughlin, K. , Lorenzetti, D. L. , Hill, M. D. , Manns, B. J. , & Ghali, W. A. (2007). Early risk of stroke after transient ischemic attack: A systematic review and meta‐analysis. Archives of Internal Medicine, 167, 2417–2422. 18071162 10.1001/archinte.167.22.2417

[brb3865-bib-0046] Yousufuddin, M. , Bartley, A. C. , Alsawas, M. , Sheely, H. L. , Shultz, J. , Takahashi, P. Y. , … Murad, M. H. (2017). Impact of multiple chronic conditions in patients hospitalized with stroke and transient ischemic attack. Journal of Stroke and Cerebrovascular Diseases: the Official Journal of National Stroke Association, 26, 1239–1248. 28285088 10.1016/j.jstrokecerebrovasdis.2017.01.015

